# A Neuroeconomics Analysis of Investment Process with Money Flow Information: The Error-Related Negativity

**DOI:** 10.1155/2015/701237

**Published:** 2015-10-18

**Authors:** Cuicui Wang, João Paulo Vieito, Qingguo Ma

**Affiliations:** ^1^School of Management, Hefei University of Technology, Hefei 230009, China; ^2^School of Business Studies, Polytechnic Institute of Viana do Castelo, 4920311 Viana do Castelo, Portugal; ^3^School of Management, Zhejiang University, Hangzhou 310058, China; ^4^Neuromanagement Lab, Zhejiang University, Hangzhou 310058, China

## Abstract

This investigation is among the first ones to analyze the neural basis of an investment process with money flow information of financial market, using a simplified task where volunteers had to choose to buy or not to buy stocks based on the display of positive or negative money flow information. After choosing “to buy” or “not to buy,” participants were presented with feedback. At the same time, event-related potentials (ERPs) were used to record investor's brain activity and capture the event-related negativity (ERN) and feedback-related negativity (FRN) components. The results of ERN suggested that there might be a higher risk and more conflict when buying stocks with negative net money flow information than positive net money flow information, and the inverse was also true for the “not to buy” stocks option. The FRN component evoked by the bad outcome of a decision was more negative than that by the good outcome, which reflected the difference between the values of the actual and expected outcome. From the research, we could further understand how investors perceived money flow information of financial market and the neural cognitive effect in investment process.

## 1. Introduction

Most of the part of the literature that analyzes the relationship between investment behavior and stock market information has been done based on historical data, such as shares you own or stock gains; however, with this information alone, it is difficult to understand how an investor acts before and during the investment decision process. One may only understand what happened after the decision was made. Recently, various groups of researchers from the areas of economics, finance, and neuroscience worked together to attempt to understand how the human brain reacts when people make financial decisions [1, 2]. Moreover, with multidisciplinary knowledge and technique, we can further understand how investors perceive kinds of information in financial market. This paper is the first step to understand how money flow information is cognized and processed during investment with technique of neuroscience.

*The Use of Neuroscience in Financial Market Investment*. Neuroscience can use functional imaging of brain activity (such as functional magnetic resonance imaging (FMRI)) and other techniques (such as electroencephalogram (EEG)) to infer details about how the brain works during financial risk decision-making. Kuhnen and Knutson (2005) used FMRI to examine anticipatory neural activity in a financial decision-making task and found that the activation of the nucleus accumbens, which is a brain region, preceded risky choices as well as risk-seeking mistakes, while the anterior insula was activated prior to riskless choices as well as risk-aversion mistakes [3]. Moreover, the anterior cingulate cortex (ACC) was involved in risk assessment and in many other higher-order cognitive functions [4, 5]. Apart from various functional imaging techniques, EEG measurement is another technique which has the cost efficient advantage and excellent temporal (timing) resolution. Event-related potentials (ERPs) are voltage fluctuations within EEG recordings which are time sequenced to specific events. Hewig et al. (2007), when investigating ERPs, found that the component of error-related negativity (ERN), one of the components which can reflect the cognitive process by recording brain evoked potentials and average superposition, was related to the degree of reward expectation, the amplitude of which was directly related to risk-taking and decision-making behavior [6]. Hajcak et al. (2006) also stated that the feedback-related negativity (FRN) component was related to the outcome of risk decision-making, which reflected the early appraisal of feedback based on a binary classification of good versus bad outcomes [7].

Although many investigations have been done in the area of decision-making under risk, few have analyzed the brain's electrical activity during realistic investment process. In this investigation, electroencephalogram technology was used to investigate behaviors and the electrical activity of investors' brain by displaying net money flow information of stocks. Money flow is an index measuring the strength of money going in and out of a stock or security in a period. Money flow index (MFI) is an important indicator for technical analysis which would help investors predict trend reversals and share price fluctuations [8]. Moreover, Gryc [9] chose MFI as one input for neural networks in predicting stock price fluctuations of companies. Net money flow (NMF) is calculated by the following formula:

(1) NMF∑MFpositive−MFnegative=∑Phigh×Volume−∑Plow×Volume,

where MF_positive_ is the positive money flow, MF_negative_ is the negative money flow, *P*_high_ and *P*_low_ are the prices of stocks when they are purchased at a higher price and lower price, respectively, and Volume is the number of shares purchased. Thus, there are two categories of net money flow: positive and negative. With this information, we investigated how a positive and/or negative net money flow was perceived and influenced the decision to buy or not to buy the stock, including the choices (the percentage of choosing “to buy” or “not to buy”), the time spent, and brain activities.

*ERP Components Involved in Investment*. One of the most commonly researched components related to risk decision-making is the ERN, which is a medial frontal negative component of an ERP, first described as a negative deflection response to errors in reaction time tasks by Falkenstein et al. (1991) and Gehring et al. (1993) [10, 11]. Larger ERNs were related to a decrease in error force, an increase in the likelihood of error correction, and slower responses in the following trial. They suggested that remedial action was one of the functions of the ERN process [11].

Holroyd et al. (2004) found that the relative outcome of the trial in relation to different possible outcomes mainly determined the amplitude of the ERN rather than the absolute outcome [12]. It corroborated the view that the relative or subjective relevance of the feedback moderated the amplitude of the ERN and a negative deviation from a reward expectation resulted in an ERN. In a gambling task where participants had to decide between larger and smaller amounts of money which turned out to be either wins or losses, ERN amplitude correlated with the differences in risk-taking behavior after monetary losses [13]. The above finding might suggest a role for the ERN in strategic adjustments of behavior and decision-making.

The literature suggests several theories about ERN, which has some controversies. One is an error detection theory, which assumes that ERN is a neural correlate of mismatch detected by comparing representations of the intended and the actual performed actions [10, 11, 14]. Another theory is a reinforcement learning theory, the basal ganglia of which continuously evaluates the outcome of ongoing behaviors against participants' expectations, and ERN plays a role in the strategic adjustment of behavior and decision-making [15, 16]. Furthermore, conflict monitoring theory assumes that ERN reflects the monitoring of response conflict during response selection [13, 16, 17]. The activation of the correct response gives rise to a transient period during which both the correct response tendency and the already executed incorrect response are activated [4].

Previous researches provided evidence for conflict monitoring theory of ERN by gambling tasks [4, 6]. Based on the blackjack gambling task, Hewig et al. (2007) found that, compared with the condition in which participants had cards with lower scores (<17), the hit decision evoked a more negative ERN for the condition in which the participants had cards with higher scores (>16) [6]. The hit decision when the cards had higher scores had more risk, and the conflict between the desire to win and the desire to be safe should be more severe. A recent study with a gambling task (where the participants who were required to choose to bet or not in each trial were presented with gain or loss feedback after each decision) observed that the ERN magnitudes were more negative for the “to bet” than for the “not to bet” choices, for both large and small stakes and choices involving large rather than small stakes were more negative [4]. It was supported that when the stakes were larger, the conflict to “to bet” was more severe. Since investment decision-making was similar to risk decision-making of a gamble, we speculated that if ERN was evoked in our experiment, conflict monitoring theory would give explanations for ERN component.

In addition to the ERN effect, there are two other components related with evaluating the outcome or feedback of behavioral performance. The first is feedback-related negativity (FRN), a negative ERP component occurring at 200–300 ms after the presentation of feedback, which is maximal over medial frontal scalp [18]. Another component is P300, which is a positive wave usually with peak occurring at about 300 ms poststimulus [19]. Yeung et al. used monetary gambling tasks and found that FRN was elicited by negative outcomes [20], while Hajcak et al. observed that larger P300 amplitudes were evoked by positive outcomes than negative outcomes [21].

This study aimed to use ERP and behavior data to provide further understanding of the cognitive processes involved in investment with money flow information. In this experiment, according to money flow information, participants were required to decide whether to buy the stock or not. Stocks with negative money flows are normally perceived as being riskier and those with positive money flow less risky. Based on this assumption, one would expect different brain reaction times when volunteers make the decision “to buy” or “not to buy” in the different contexts. The literature citing the above suggests that ERN might reflect the level of riskiness of different choices. Accordingly, one would suggest that ERN could be found to reflect the investment decision process. Additionally, there was a feedback after each decision in this experiment, and FRN and P300 would be elicited by evaluating different kinds of feedbacks.

## 2. Materials and Methods

### 2.1. Participants

Twenty-nine major undergraduates (15 females, all right-handed) aged from 20 to 26 years (mean age, 23.5 years) from Zhejiang University participated in this experiment as paid volunteers. They were all familiar with stock investment and had some knowledge on money flow in stock markets but no experience of real investment. They were all native Chinese speakers and had normal or corrected to normal vision with no history of neurological or psychiatric abnormalities. There were only twenty-seven participants recording behavioral data, as some problems were found in the software codes and the behavioral data of the first two participants were not recorded. Before experiment, informed consent was obtained from all volunteers, and the research was approved by the Internal Review Board of Zhejiang University Neuromanagement Lab.

### 2.2. Electroencephalogram Recording

The experiment was performed in an electrically shielded and sound-attenuated cabin. Participants sat in a comfortable chair and a computer display was located 1 m away from their eyes. The electroencephalography (EEG) was recorded (band pass 0.05–100 Hz, sampling rate 500 Hz) with Neuroscan Synamp2 Amplifier (Scan 4.3.1, Neurosoft Labs Inc.), using Ag/AgCl electrodes placed at 64 scalp sites according to the extended international 10–20 system and referenced to left mastoid with a cephalic (forehead) location as ground. Vertical electrooculograms (EOG) were recorded with one pair of electrodes placed above and below the left eye, horizontal EOG with another pair 10 mm from the lateral canthi. Electrode impedances were maintained below 5 kΩ throughout the experiment. Before the formal experiment was presented, participants were given written and oral instructions and then practiced 30 trial runs.

### 2.3. Experimental Stimuli

The ERP was measured using a simplified investment task, in which participants were asked to make decisions on hundreds of stocks with net money flow information and then given the feedback. Net money flow as stimuli 1 (S1) contained two categories: positive net money flow information and negative net money flow information. Feedback as stimuli 2 (S2) included three categories: increase, decrease, and no significant change. In order to exclude other factors and keep ERP experiment simple, S1 and S2 only referred to the data of real financial market, but participants were not told the truth.

Information of S1 used referred to money flow information on stocks from June and July (2011), the prices of which were between 18 Chinese Yuan (CNY) (about $2.89) and 22 CNY (about $3.53), with an average of 20 CNY (about $3.2). By ranking the stock according to the absolute value of the net money flow, it was found that the absolute value of the data which came in the first twenty varied greatly. The absolute value of 40 million to 60 million (CNY) was obtained from questionnaires which had also been administered to participants and this was large enough for nonexperienced participants. Therefore, ten figures as positive data from 40 million and 60 million (CNY) and ten figures as negative data from −60 million to −40 million (CNY) were chosen (see Table 1) as net money flow information for S1.

The S2 consisted of thirty pictures reflecting the increase/decrease of stock in percentage terms, divided into three categories: the percentage close to zero, the positive percentage between 8% and 10%, and the negative percentage between −10% and −8% (ten pictures per category, see Table 1). In order to distinguish positive and negative percentage data, positive feedbacks were highlighted in red and negative in green (in tune with real market). Each picture was digitized at 400 × 300 pixels with a gray background.

### 2.4. Experiment Procedure

The stimuli consisted of 600 pairs of stimuli (S1) – feedback stimuli (S2), that is, 10 figures (reflecting the net money flow of stocks) × 2 categories (negative or positive) × 10 feedback figures × 3 categories (increase and decrease, no significant change). They were presented in the center of a screen with black words for the S1 and red or green words for the S2 on a grey background. E-prime software was employed to control the presentation of the stimuli in a random manner.

**Figure 1:**
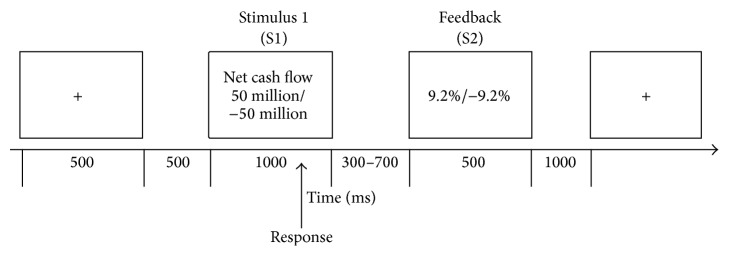
ERP paradigm.

The experiment was composed of five blocks. For each trial of a block, firstly, there was a “+” presented for 500 millisecond (ms) at the beginning; then, S1 was presented for 1000 ms followed by a random interstimulus interval between S1 and S2, ranging from 300 ms to 700 ms (average mean was 500 ms). The feedback stimuli (S2) was presented for 500 ms, and the interval between the end of S2 and the onset of “+” was 1000 ms (see Figure 1).

Before the experiment, participants were asked to fill in a questionnaire, which was used to assess their familiarity with stock investment and net money flow. Participants were informed that we chose 600 different stocks from the real stock market, the prices of which were approximately 20 CNY in June or July 2011. The net money flow figures (S1) of all stocks were chosen after the morning trading one day in June or July 2011 from a real stock market. The feedback (S2) represented the real increased percentage of stocks following the closing on that day. Throughout each trial, they had to focus on the stimuli and buy one hundred shares or not, according to the net money flow (S1) stimuli. The objective was to obtain the best results from to buy or not to buy stock, as this would result in a monetary reward. Half of the participants were instructed “if buy, please press “1” with left hand; otherwise press “3” with the right,” and the others were instructed to press “3” with right hand “if you buy”; otherwise press “1” with the left for the balance. They were told that all stocks were irrelevant and independent. In order to ensure that participants put their efforts in the experiment, there was an additional monetary reward paid depending on their performance, except for 15 CNY (about $2) as basic payment. After each block, we chose four trials randomly, and if participants chose to buy, the additional monetary reward took into account the share price in the end of the day (the data of feedback added 1 and multiplied by the basic price 20 CNY); otherwise the share price in the beginning (20 CNY) was taken into account. We averaged all share prices of stocks which were chosen from five blocks and gave participants as monetary reward of experiment. Additionally, participants were asked to make decision as soon as possible when the net money flow (S1) was presented.

### 2.5. Electroencephalogram Analysis

EEG recordings for ERN were extracted from −400 ms to 400 ms time-locked to the response onset, with the period from −400 ms to −200 ms prior to the response as baseline. EOG artifacts were corrected using the method proposed by [22]. Trials containing EOG activity or other artifacts (bursts of electromyographic activity or peak-to-peak deflection exceeding ±80 *μ*V) were excluded from averaging. The remaining trials were corrected to baseline. ERPs were averaged for every participant in each of the four conditions according to categories of S1 and response categories for each electrode site, that is, 2 categories of S1 (the positive net money flow and the negative net money flow) × 2 response types (to buy or not). The averaged ERPs were digitally filtered with a low pass filter at 30 Hz (24 dB/Octave).

**Figure 2:**
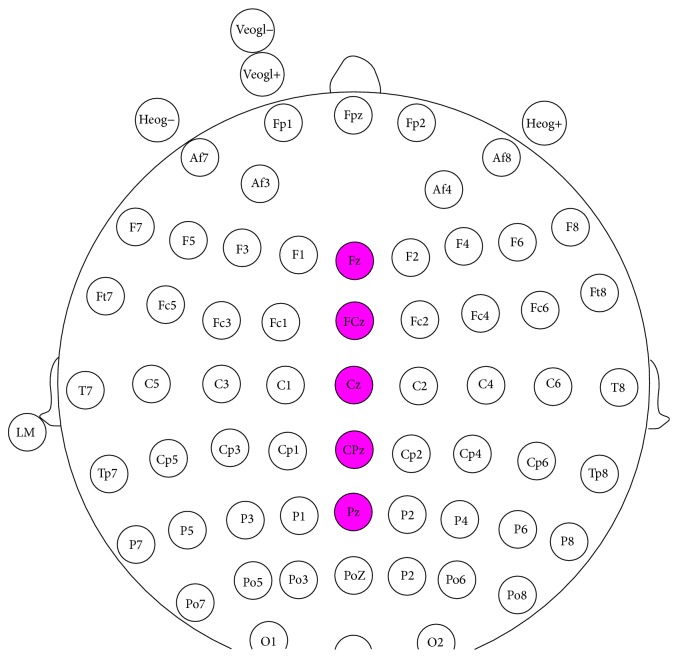
Description of brain EEG location of all the electrodes (the amplitudes of ERN and FRN were calculated for five midline pink electrodes (Fz, FCz, Cz, CPz, and Pz)).

To quantify the response-locked ERN component, a 2 (net money flow category) × 2 (response category) × 5 (electrodes) within-subjects repeated measure ANOVA for ERN was conducted. The Greenhouse and Geisser (1959) correction was applied for the violation of sphericity assumption when necessary (uncorrected* df* was reported with the *ε* and corrected *p* values), and the Bonferroni correction was used for multiple paired comparisons. For the present report, five electrodes: Fz, FCz, Cz, CPz, and Pz (see the pink electrodes in Figure 2), were used for ERN statistical analysis. The restriction of the electrode set in the ERN statistical analyses was based on the hypothesis that a frontocentral midline maximum was expected for ERN and the ERN effects were the strongest of these [6].

For the feedback (S2), electroencephalogram recordings were extracted from −200 ms to 800 ms stimulus-locked to the onset of S2, with the period from −200 ms to 0 ms as baseline. Walsh and Anderson (2012) proved that FRN arose in the anterior cingulate cortex, which was similar with ERN [23]. Many researches chose Fz, FCz, Cz, CPz, and Pz electrodes for FRN analysis [23, 24]. Based on the above information, ERP amplitudes following feedback were also calculated for five midline electrodes (Fz, FCz, Cz, CPz, and Pz, see the pink electrodes in Figure 2).

If the participant decides to “to buy,” normally they expect stock prices to rise in the future, while if the choice is “not to buy,” their expectation is that the stock price will not rise in the future (it is better to drop). As such, the outcome of the decisions was classified into three categories: good feedback (the feedback was identical with expectation), bad feedback (the feedback was opposite to expectation), and constant feedback (the feedback had no significant change). Following that, a 3 (feedback categories) × 5 (electrodes) within-subjects repeated measure ANOVA for feedback stimuli was conducted.

Behavioral measures (choices in percentage and reaction times) were also considered via an analysis of variance (ANOVA) with response types (“to buy” or “not to buy”) and the categories of net money flow (positive or negative) as two within-participant factors.

## 3. Results

### 3.1. Behavioral Data

**Figure 3:**
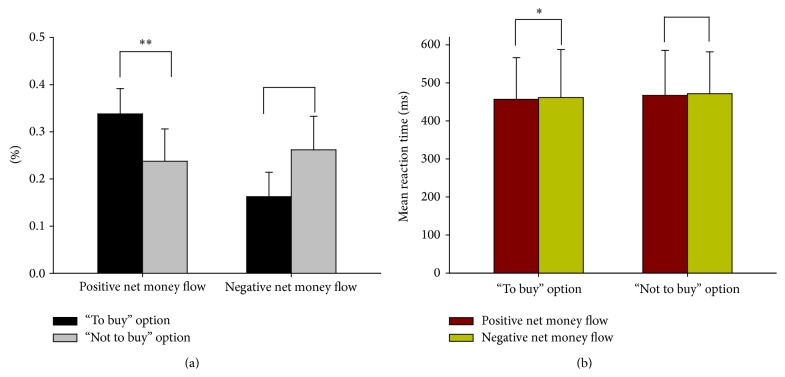
(a) Comparing “to buy” option and “not to buy” option in percentage in positive and negative net money flow category, respectively. Error bars indicate SD of choices in percentage. (b) Mean RTs of positive and negative net money flow sorted by response “to buy” and “not to buy,” respectively. Error bars indicate SD of RT.

The statistical results for the choices in percentage and for the reaction times (RTs) in four conditions were presented in Figure 3.

For percentage of choices, there was a significant difference in the response type factor (*F*(1, 26) = 16.262, *p* = 0.000), and the percentage of “to buy” (M = 0.288, SD = 0.009) was larger than that of “not to buy” (mean = 0.212, SD = 0.009). Therefore, this would suggest that the participants in our experiment were more risk taking. There was a significant interaction between the net money flow and response type factors (*F*(1, 26) = 46.622, *p* = 0.000), but with no difference between choices in the net money flow factor (*F*(1, 26) = 0.151, *p* = 0.701). In the positive net money flow category, there was a significant difference in percentage between the “to buy” and “not to buy” choices (*F*(1, 26) = 74.765, *p* = 0.000) and the percentage of choices “to buy” (M = 0.338, SD = 0.010) was greater than that of “not to buy” (M = 0.162, SD = 0.010). However, no significant difference was found in the category of negative money flow.

For RTs, there was a significant difference in the net money flow factor (*F*(1, 26) = 9.098, *p* = 0.006), however, with no significant difference in the factor of response types (*F*(1, 26) = 0.769, *p* = 0.389). It suggested that participants responded faster in the condition of positive net money flow (M = 459.07 ms, SD = 118.97) than in the condition of negative net money flow (M = 469.35 ms, SD = 115.31). There was a significant difference when the RTs of “to buy” in the two categories of the net money flow were compared (*F*(1, 26) = 5.211, *p* = 0.031). It was faster for the RTs of “to buy” in the positive net money flow than in the negative net money flow. However, no significant difference was found when comparing the RTs of “not to buy” in the two categories of the net money flow (*F*(1, 26) = 4.480, *p* = 0.037). The ANOVA revealed that the RTs had no significant interaction with the factors of net money flow and response types (*F*(1, 26) = 0.000, *p* = 0.997).

### 3.2. ERP Data

#### 3.2.1. ERP Data of Stimuli (S1)

**Figure 4:**
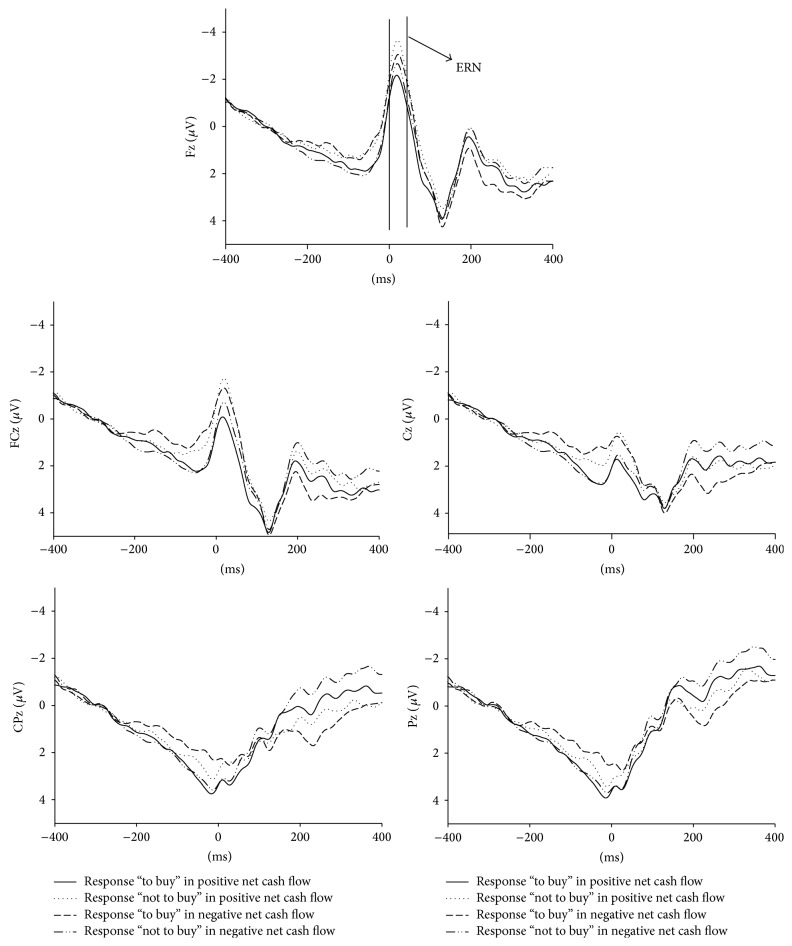
The grand averaged ERPs of S1 from channels Fz, FCz, Cz, CPz, and Pz are described separately for the “to buy” response in the positive net money flow, “not to buy” response in the positive net money flow, “to buy” response in the negative net money flow, and “not to buy” response in the negative net money flow.

The response-locked grand average ERPs of S1 for “to buy” response in the positive net money flow, “not to buy” response in positive net money flow, “to buy” response in the negative net money flow, and “not to buy” in the negative net money flow were displayed in Figure 4. Response onset was presented at 0 msec. From the figure, we can see the negative net money flow evoked significantly larger negative but less negative amplitude than positive net money flow for ERN in the responses “to buy” and “not to buy,” respectively.

The ANOVA for the mean amplitude of ERN in the 0 to 40 ms time window revealed no significant effects for the net money flow (*F*(1, 28) = 0.931, *p* = 0.343) and response type factors (*F*(1, 28) = 2.859, *p* = 0.102). However, significant effects for the electrodes factor were detected (*F*(4, 112) = 80.890, *ε* = 0.479, *p* = 0.000). For the amplitude of electrodes, the mean negative voltages distributed from frontal to parietal regions showed a decline trend: frontal electrode Fz (M = −2.665 *μ*V, SD = 0.627), frontal-central electrode FCz (M = −0.812 *μ*V, SD = 0.738), central electrode Cz (M = 1.161 *μ*V, SD = 0.763), central-partial electrode CPz (M = 2.722 *μ*V, SD = 0.706), and partial electrode Pz (M = 2.958 *μ*V, SD = 0.627). There were significant differences among each electrode except that there was no significant difference between the CPz and Pz electrodes.

The ANOVA revealed that the ERN had significant interaction with the net money flow and response type factors (*F*(1, 28) = 10.810, *p* = 0.003). However, there was no significant interaction with the net money flow and electrodes factors, or the response type and electrodes, or the net money flow × response type × electrodes. Furthermore, for ERN in the responses “to buy,” the negative net money flow (M = 0.521, SD = 0.664) evoked significantly larger negative amplitude than positive net money flow (M = 1.512, SD = 0.704) (*F*(1, 28) = 7.294, *p* = 0.012), while, for ERN in the responses “not to buy,” the positive net money flow (M = −0.390, SD = 0.848) evoked significantly larger negative amplitude than negative net money flow (M = 1.048, SD = 0.669) (*F*(1, 28) = 8.406, *p* = 0.007).  Table 2 showed all the statistical results of within-subjects repeated measure ANOVA for ERN.

#### 3.2.2. Feedback Stimuli (S2)

**Figure 5:**
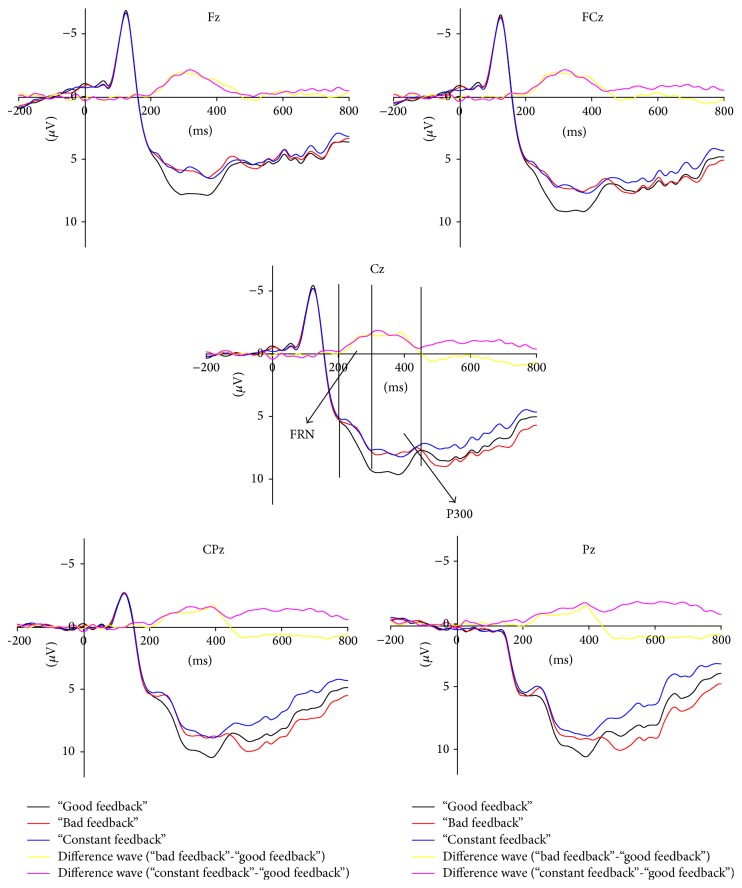
The grand averaged ERPs of S2 from channels Fz, FCz, Cz, CPz, and Pz separately for the “good feedback,” “band feedback,” and “constant feedback.”

For feedback stimuli, the grand average for the Fz, FCz, Cz, CPz, and Pz electrodes is shown in Figure 5. Feedback stimuli onset was presented at 0 msec. In stimulus-locked ERPs analysis, −200 ms to 0 ms was chosen as the baseline. From this figure, the amplitude of FRN and P300 evoked by “bad feedback” and “constant feedback” was more negative than “good feedback.”

In the case of “bad feedback,” it is possible to observe that the FRN appeared to peak at approximately 250 ms after the feedback stimulus. The ANOVA for the mean amplitude of FRN in the 200 to 300 ms time window was conducted, and the main effect of feedback categories (good feedback versus bad feedback versus constant feedback) was found (*F*(2, 56) = 12.111, *p* = 0.000) with no significant effect of electrode location. By pairwise comparisons, the amplitude evoked by “bad feedback” (*p* = 0.005) and “constant feedback” (*p* = 0.000) was more negative than that evoked by “good feedback.” However, there was no significant difference between “bad feedback” and “constant feedback” (*p* = 1).

In the time window of 300 ms to 450 ms after the feedback presentation for P300, there were main significant effects in the feedback category (*F*(2, 56) = 18.506, *p* = 0.000) and electrode location (*F*(4, 112) = 10.106, *ε* = 0.354, *p* = 0.000). By pairwise comparisons, the amplitude evoked by “bad feedback” (*p* = 0.000) and “constant feedback” (*p* = 0.000) was more negative than that evoked by “good feedback.” However, there was no significant difference between “bad feedback” and “constant feedback” (*p* = 0.723).

## 4. Discussion

In this experiment, we used ERPs to examine the perception of money flow information and further understand the cognitive processes involved in investment. It was observed that ERN components were more negative for the “to buy” option but were less negative for the “not to buy” option in the negative net money flow than in the positive net money flow. We suggested that the factor of net money flow affected the risk level of stocks and the findings for the ERN effect in the investment decision process were sensitive to the risk evaluation of the choices. ERN may be used to act as an alerting system to prepare the brain for the potential negative consequences of actions. For the FRN component, actual results that were different from expectations evoked more negative amplitude than those that were in line with expectations, reflecting the different cognitions for different kinds of feedback results.

The behavioral data which demonstrated that participants were more likely to choose “to buy” than “not to buy” reflected risk taking, which was consistent with previous investigations [4, 6]. Compared with the choices in the negative net money flow condition, there was larger percentage of “to buy” option and less percentage of “not to buy” option in the positive net money flow condition. As a positive net money flow condition of the stock was regarded as having a larger possibility of good performance in the future which may be considered low risk, the conflict to choose “not to buy” was severe; however, the same was true for the negative net money flow condition. This was also supported by the RTs results. Compared to the RTs in the negative net money flow, it was easier and implied significantly shorter response time to choose the “to buy” option in the positive net money flow.

Clearly, the amplitude of ERN evoked by the decision “to buy” was significantly more negative in the negative net money flow than in the positive net money flow; however, ERN elicited by the decision “not to buy” had an inverse effect. The component of ERN reflected the neural basis in the investment decision process with money flow information. Decision-making on investment referred to the process by which one action (e.g., “to buy” or “not to buy”) was chosen based on the assessment of the potential costs and benefits associated with it. In this experiment, participants were paid according to the outcome of their choices; therefore, they had to compare risk with reward, choose one action, and evaluate the outcome obtained for that particular action. In real stock markets, we only know that investors refer to money flow information in investment decision-making, with no profound understanding of its effect and the cognitive process. We speculated from ERN results that stocks with larger positive net money flow were often considered as low-risk stocks and had a stronger possibility to increase. However, stocks with large negative net money flow were often considered as high-risk stock and had a greater probability to decrease in price. For the purpose of earning greater returns, the conflict to choose “to buy” in positive net money flows was smaller than in negative net money flows; however, this decision was severer for the “not to buy” option. The findings provided further evidence that conflict monitoring theory was one important function of ERN component.

In this investigation, the amplitude of ERN was more negative in the frontal, frontal-central, and central areas than in the central partial and partial areas. This may be related to the source of the ERN. Many investigations by dipole source analysis of the ERN, which can speculate the source of the ERN components according to the EEG scalp distribution, suggested that this component was generated in the anterior cingulate cortex (ACC) [25–27]. The ACC is connected to the areas of the brain which are responsible for drive and arousal, for motor output and for cognition, and which deal with risk assessment [28–30]. According to an extended version of the conflict monitoring theory [31], the monitored ACC is activated not only during different stimulus-response mappings in the selection of the responses, but also when different internal desires are aroused [4]. During the investment with different money flow information, the corresponding levels of desire to win and desire to be safe with action “to buy” and “not to buy” were not the same. And the conflict of taking a risk when choosing “to buy” in a negative net money flow or rejecting by choosing “not to buy” in a positive net money flow may well be detected by the ACC and the ERN effect was thus ensured.

FRN follows the display of negative feedback [32]. It can be used as a tool for studying reward valuation and decision-making, which reflects the difference between the values of actual and expected feedback or reward prediction errors [23, 33]. In this experiment, when the participant chose to buy the stock in question, their expectation was that the price of the stock would increase, and when the participant chose not to buy the stock, they hoped the price of the stock would not rise. If the feedback was not in line with their predictions, FRN was evoked. There was no difference between “bad feedback” and “constant feedback” in the amplitude of FRN, but there was a significant difference between “good feedback” and “bad feedback.” The results may show that the effect derived from the stock for which prices did not change was the same as that for stock for which the prices dropped in the mind of the investors. However, in the later time window of 300 ms to 450 ms, the finding of P300 increased amplitudes for “good feedback” reflected different emotional state to feedback stimuli. Previous research found that P300 amplitude was larger for emotional stimuli than for neural stimuli [33]. Furthermore, the positive emotional stimuli induced larger P300 than the negative stimuli [34]. In this study, “good feedback” induced a positive emotional state, but “bad feedback” or “constant feedback” made investors have a bad mood.

There were some differences between ERN and FRN evoked in our experiment and traditional ERN and FRN. Traditionally, the ERN peaks 100 ms after response errors, and FRN emerges at 200 ms and peaks 300 ms after negative feedback onset [23]. However, in our study, the ERN peaked around 20 ms after response onset, and FRN also emerged at 200 ms but with peak of about 250 ms after negative feedback (bad feedback or constant feedback). It is because our paradigm was more complex than the usual paradigms used for ERN and FRN research. Typical ERN appears in speeded reaction tasks, in which errors are due to impulsive responding. But, in our experiment, it was a more complicated cognitive process under investment with money flow information. ERN was evoked not by error response but by conflict/risk perception. For FRN in our experiment, we speculated that as the feedback was closely connected with decision behavior of participants, the feedback stimuli may be processed into cognition earlier and the latency of FRN was less than traditional latency (300 ms).

One might ask why there was no significant difference between the choices of “to buy” and “not to buy” in a negative net money flow and why there was no significantly different effect in the ERN between response of “to buy” and “not to buy” in a negative net money flow. The main reason was that people preferred to take risk. In the gambling tasks, Hajcak et al. (2006) and Yu and Zhou (2009) found that people were more likely to choose “to buy” than “not to buy” in risk decision [4, 7]. This result was also supported by the data from the experiment which demonstrated that the percentage of participants choosing “to buy” was larger than that of those choosing “not to buy.” However, in the negative net money flow, data suggested that participants experienced some conflict when deciding to buy with higher risks, and this to some extent inhibited participants' fondness for taking risk. Therefore, the number of choices “to buy” may decrease but there was no significant difference with the “not to buy” choices. This may explain why no significant difference was found in the ERN amplitude between the “to buy” and “not to buy” responses in a negative net money flow.

Findings reported in this paper were a good starting point to further understand the cognitive processes in investment behavior of financial market. However, real financial market is a complex environment in which there are so many factors (such as industry information, government policy, and other persons' behavior) affecting the investment of decision-making. The lab studies using EEG or FMRI method only focus on one or two factors and control other factors. As our study only aimed at the role of money flow information in investment, further research should investigate how other factors of financial market affect the cognitive process of investment. Moreover, our study only recruited college students with stock knowledge but no speculation experience, and in further research we can study whether the neural basis of investment could be modulated by individual investing experience. Although more refining and simplifying of the paradigm used in this paper was required, it is a small forward step to use basic neuroscience research methods and apply them to real world practice.

The paper used EEG technique to infer details about how the brain works during financial risk decision-making, which provided the new insights into the financial computation and financial intelligence analysis. In financial decision-making, many models posited distinct cognitive and emotional contributions to decision-making under uncertainty, and cognitive processes typically involved exact computations according to a cost-benefit calculus [35]. This paper could provide a new reference for the cognitive processes in the financial computation based on EEG signals.

## 5. Conclusion

Using a simplified investment task in which a participant had to choose to buy or not to buy one stock with net money flow information, it was demonstrated that the ERN was more negative for the “to buy” response but less negative for the “not to buy” response in the negative net money flow than in the positive net money flow. FRN was larger when evoked by “bad feedback” than when evoked by a “good feedback,” which reflected the difference between the values of actual and expected outcomes. From this experiment, we could further understand the neural cognitive effect of money flow information in the process of investment. The ERN which was generated by ACC reflected the function of conflict monitoring in risk investment. This component may be used as an early warning index to alert the brain to prepare for potential negative consequences when investing in high-risk stock or other risky actions.

## Figures and Tables

**Table 1:** The experimental stimuli of net money flow (S1) and the feedback (S2).

Positive net money flow (S1)	Negative net money flow (S1)	Increase (S2)	Decrease (S2)	No significant change (S2)
40 million	−40 million	+8.0%	−8.0%	+0.2%
42 million	−42 million	+8.2%	−8.2%	+0.4%
44 million	−44 million	+8.4%	−8.4%	+0.6%
46 million	−46 million	+8.6%	−8.6%	+0.8%
48 million	−48 million	+8.8%	−8.8%	+1.0%
50 million	−50 million	+9.0%	−9.0%	−0.2%
52 million	−52 million	+9.2%	−9.2%	−0.4%
54 million	−54 million	+9.4%	−9.4%	−0.6%
56 million	−56 million	+9.6%	−9.6%	−0.8%
58 million	−58 million	+9.8%	−9.8%	−1.0%

**Table 2:** The statistical results of within-subjects repeated measure ANOVA for ERN with factors Net money flow (positive/negative), response type (“to buy”/“not to buy”), and electrodes (Fz, FCz, Cz, CPz, and Pz).

Factor	*df*, error	Greenhouse and Geisser (*ε*)	*F*/*t* value	*p* value
Main effect				
Net money flow	1, 28	—	0.931	0.343
Response type	1, 28	—	2.859	0.102
Electrodes	4, 112	0.478	80.89	**0**
Interaction effect				
Interaction of net money flow and response type	1, 28	—	10.81	**0.003**
Interaction of net money flow and electrodes	4, 112	0.481	0.794	0.453
Interaction of response type and electrodes	4, 112	0.533	2.6	0.079
Interaction of net money response, response type, and electrodes	4, 112	0.415	1.166	0.33
Simple effect				
Net money flow in response of “to buy”	1, 28	—	7.294	**0.012**
Net money flow in response of “not to buy”	1, 28	—	8.406	**0.007**

## References

[1] Hubert M. (2010). Does neuroeconomics give new impetus to economic and consumer research?. *Journal of Economic Psychology*.

[2] Witt U., Binder M. (2013). Disentangling motivational and experiential aspects of ‘utility’—a neuroeconomics perspective. *Journal of Economic Psychology*.

[3] Kuhnen C. M., Knutson B. (2005). The neural basis of financial risk taking. *Neuron*.

[4] Yu R., Zhou X. (2009). To bet or not to bet? The error negativity or error-related negativity associated with risk-taking choices. *Journal of Cognitive Neuroscience*.

[5] van Duijvenvoorde A. C. K., Huizenga H. M., Somerville L. H. (2015). Neural correlates of expected risks and returns in risky choice across development. *Journal of Neuroscience*.

[6] Hewig J., Trippe R., Hecht H., Coles M. G. H., Holroyd C. B., Miltner W. H. R. (2007). Decision-making in blackjack: an electrophysiological analysis. *Cerebral Cortex*.

[7] Hajcak G., Moser J. S., Holroyd C. B., Simons R. F. (2006). The feedback-related negativity reflects the binary evaluation of good versus bad outcomes. *Biological Psychology*.

[8] Brock W., Lakonishok J., LeBaron B. (1992). Simple technical trading rules and the stochastic properties of stock returns. *The Journal of Finance*.

[9] Gryc W. (2010). Neural network predictions of stock price fluctuations. http://i2r.org/nnstocks.pdf.

[10] Falkenstein M., Hohnsbein J., Hoormann J., Blanke L. (1991). Effects of crossmodal divided attention on late ERP components. II. Error processing in choice reaction tasks. *Electroencephalography and Clinical Neurophysiology*.

[11] Gehring W. J., Goss B., Coles M. G., Meyer D. E., Donchin E. (1993). A neural system for error detection and compensation. *Psychological Science*.

[12] Holroyd C. B., Larsen J. T., Cohen J. D. (2004). Context dependence of the event-related brain potential associated with reward and punishment. *Psychophysiology*.

[13] Yeung N., Sanfey A. G. (2004). Independent coding of reward magnitude and valence in the human brain. *The Journal of Neuroscience*.

[14] Coles M. G. H., Scheffers M. K., Holroyd C. B. (2001). Why is there an ERN/Ne on correct trials? Response representations, stimulus-related components, and the theory of error-processing. *Biological Psychology*.

[15] Holroyd C. B., Coles M. G. H. (2002). The neural basis of human error processing: reinforcement learning, dopamine, and the error-related negativity. *Psychological Review*.

[16] Holroyd C. B., Yeung N., Coles M. G. H., Cohen J. D. (2005). A mechanism for error detection in speeded response time tasks. *Journal of Experimental Psychology: General*.

[17] Botvinick M. M., Carter C. S., Braver T. S., Barch D. M., Cohen J. D. (2001). Conflict monitoring and cognitive control. *Psychological Review*.

[18] Yu R., Luo Y., Ye Z., Zhou X. (2007). Does the FRN in brain potentials reflect motivational/affective consequence of outcome evaluation?. *Progress in Natural Science*.

[19] Sutton S., Braren M., Zubin J., John E. R. (1965). Evoked-potential correlates of stimulus uncertainty. *Science*.

[20] Yeung N., Holroyd C. B., Cohen J. D. (2005). ERP correlates of feedback and reward processing in the presence and absence of response choice. *Cerebral Cortex*.

[21] Hajcak G., Holroyd C. B., Moser J. S., Simons R. F. (2005). Brain potentials associated with expected and unexpected good and bad outcomes. *Psychophysiology*.

[22] Semlitsch H. V., Anderer P., Schuster P., Presslich O. (1986). A solution for reliable and valid reduction of ocular artifacts, applied to the P300 ERP. *Psychophysiology*.

[23] Walsh M. M., Anderson J. R. (2012). Learning from experience: event-related potential correlates of reward processing, neural adaptation, and behavioral choice. *Neuroscience and Biobehavioral Reviews*.

[24] Potts G. F., Martin L. E., Kamp S.-M., Donchin E. (2011). Neural response to action and reward prediction errors: comparing the error-related negativity to behavioral errors and the feedback-related negativity to reward prediction violations. *Psychophysiology*.

[25] Gehring W. J., Himle J., Nisenson L. G. (2000). Action-monitoring dysfunction in obsessive-compulsive disorder. *Psychological Science*.

[26] Miltner W. H. R., Brauer J., Hecht H., Trippe R., Coles M. G. H. (2004). Parallel brain activity for self-generated and observed errors. *Errors, Conflicts, and the Brain: Current Opinions on Performance Monitoring*.

[27] van Schie H. T., Mars R. B., Coles M. G. H., Bekkering H. (2004). Modulation of activity in medial frontal and motor cortices during error observation. *Nature Neuroscience*.

[28] Ernst M., Nelson E. E., McClure E. B. (2004). Choice selection and reward anticipation: an fMRI study. *Neuropsychologia*.

[29] McCoy A. N., Platt M. L. (2005). Risk-sensitive neurons in macaque posterior cingulate cortex. *Nature Neuroscience*.

[30] Paus T. (2001). Primate anterior cingulate cortex: where motor control, drive and cognition interface. *Nature Reviews Neuroscience*.

[31] Van Veen V., Cohen J. D., Botvinick M. M., Stenger V. A., Carter C. S. (2001). Anterior cingulate cortex, conflict monitoring, and levels of processing. *NeuroImage*.

[32] Miltner W. H. R., Braun C. H., Coles M. G. H. (1997). Event-related brain potentials following incorrect feedback in a time-estimation task: evidence for a ‘generic’ neural system for error detection. *Journal of Cognitive Neuroscience*.

[33] Bai Y., Katahira K., Ohira H. (2015). Valence-separated representation of reward prediction error in feedback-related negativity and positivity. *Neuroreport*.

[34] Johnston V. S., Miller D. R., Burleson M. H. (1986). Multiple P3s to emotional stimuli and their theoretical significance. *Psychophysiology*.

[35] Quartz S. R. (2009). Reason, emotion and decision-making: risk and reward computation with feeling. *Trends in Cognitive Sciences*.

